# Analysis of translational errors in frame-based and frameless cranial radiosurgery using an anthropomorphic phantom*

**DOI:** 10.1590/0100-3984.2015.0053

**Published:** 2016

**Authors:** Taynná Vernalha Rocha Almeida, Arno Lotar Cordova Junior, Pedro Argolo Piedade, Cintia Mara da Silva, Priscila Marins, Cristiane Maria Almeida, Gabriela R. Baseggio Brincas, Danyel Scheidegger Soboll

**Affiliations:** 1 MSc, Radiologic Technologist, Doctoral Student at the Faculdades Pequeno Príncipe, Curitiba, PR, Brazil.; 2 MD, Radiotherapist at the Centro de Radioterapia São Sebastião, Florianópolis, SC, Brazil.; 3 MD, Physicist at the Centro de Radioterapia São Sebastião, Florianópolis, SC, Brazil.; 4 Radiology Technologist, Dosimetrist at the Centro de Radioterapia São Sebastião, Florianópolis, SC, Brazil.; 5 Radiology Technologist, Masters Student at the Universidade Tecnológica Federal do Paraná (UTFPR), Curitiba, PR, Brazil.; 6 MD, Imaging Physicist at the Centro de Diagnóstico Médico Imagem, Florianópolis, SC, Brazil.; 7 PhD, Professor in the Physics Department, Universidade Tecnológica Federal do Paraná (UTFPR), Curitiba, PR, Brazil.

**Keywords:** Frame cranial radiosurgery, Frameless cranial radiosurgery, IGRT, Setup errors, Residual errors, Phantoms, imaging

## Abstract

**Objective:**

To evaluate three-dimensional translational setup errors and residual errors in image-guided radiosurgery, comparing frameless and frame-based techniques, using an anthropomorphic phantom.

**Materials and Methods:**

We initially used specific phantoms for the calibration and quality control of the image-guided system. For the hidden target test, we used an Alderson Radiation Therapy (ART)-210 anthropomorphic head phantom, into which we inserted four 5mm metal balls to simulate target treatment volumes. Computed tomography images were the taken with the head phantom properly positioned for frameless and frame-based radiosurgery.

**Results:**

For the frameless technique, the mean error magnitude was 0.22 ± 0.04 mm for setup errors and 0.14 ± 0.02 mm for residual errors, the combined uncertainty being 0.28 mm and 0.16 mm, respectively. For the frame-based technique, the mean error magnitude was 0.73 ± 0.14 mm for setup errors and 0.31 ± 0.04 mm for residual errors, the combined uncertainty being 1.15 mm and 0.63 mm, respectively.

**Conclusion:**

The mean values, standard deviations, and combined uncertainties showed no evidence of a significant differences between the two techniques when the head phantom ART-210 was used.

## Abstract (pt)

**Objetivo:**

Comparar os erros de posicionamento e erros residuais translacionais tridimensionais de uma radiocirurgia guiada por imagem, *frame versus frameless*, com uso de um objeto simulador antropomórfico.

**Materiais e Métodos:**

Para a calibração e qualidade do sistema de imagem foram utilizados objetos simuladores específicos. Para o teste *hidden target* foi utilizado o crânio do objeto simulador antropomórfico Alderson Radiation Therapy (ART)-210, dentro do qual foram inseridas quatro esferas metálicas de 5 mm de diâmetro como volumes alvos de tratamento. Imagens tomográficas foram realizadas com o ART-210 devidamente posicionado para ambos os métodos de imobilização.

**Resultados:**

Para o método *frameless*, a média foi 0,22 ± 0,04 mm para os erros *setup* e 0,14 ± 0,02 mm para os erros residuais, apresentando uma incerteza combinada de 0,28 mm e 0,16 mm, respectivamente. Para o método *frame*, a média foi 0,73 ± 0,14 mm para os erros *setup* e 0,31 ± 0,04 mm para os erros residuais, apresentando uma incerteza combinada de 1,15 mm e 0,63 mm, respectivamente.

**Conclusão:**

Com base nas médias, desvios-padrão e incertezas combinadas, os resultados mostraram não haver evidências de diferença significativa entre as técnicas em questão quando utilizado um objeto simulador antropomórfico craniano ART-210.

## INTRODUCTION

The current literature suggests that cranial radiosurgery, a high-precision radiotherapy technique to treat benign and malignant lesions, is as efficient as invasive procedures for tumors of up to 3 cm^(1)^. Traditionally, this technique requires the use of a stereotactic arc with bone fixation properly positioned by a neurosurgeon after local anesthesia^(2)^. This type of procedure is referred to as frame-based radiosurgery. It is a well-established system in the literature with minimum possibility for cranial movement and is therefore considered the gold standard in radiosurgery^(3)^.

The development of new radiotherapy methods, including image-guided radiotherapy (IGRT), has led to a new noninvasive immobilization system, referred to as frameless radiosurgery. In this system, a set of thermoplastic masks is used in order to mold the cranial surface of the patient^(4)^.

In IGRT, variations in positioning between the planning and execution of treatment are referred to as setup errors. The system identifies these errors and corrects them, allowing for a reduction in values, resulting in errors that are within acceptable limits, known as residual errors.

The performance, precision, and accuracy with which the equipment in a radiotherapy system deliver the radiation dose to a previously detected lesion depend on the results of the quality control tests^(5)^. Those tests usually need appropriate phantoms in order to be carried out successfully.

In order to broaden the information available on the precision of the different immobilization methods in radiosurgery, this study aims to compare setup errors and three-dimensional (3D) translational residual errors in image-guided radiosurgery, for frame and frameless methods, using the endto-end test, with a head and neck phantom (Rando ART-210; Alderson Research Laboratories, Long Beach, CA, USA).

## MATERIALS AND METHODS

### ExacTrac 5. 5 X-ray 6D system for intracranial stereotactic radiosurgery

The ExacTrac system (Brainlab AG, Feldkirchen, Germany) uses optical tracking of reflective spheres and X-ray records to determine and correct patient positioning in real time. This system includes a camera that can transmit and receive infrared signals, with which the optical tracking is possible, together with double-assembly kV energy X-ray tubes and silicon detectors, which generate orthogonal images and merge X-ray images with planning computed tomography (CT) images, using digital reconstructed radiography^(6)^.

### System quality control and calibration tests

To improve the precision of the infrared camera and X-ray tubes, the ExacTrac isocenter must be calibrated and checked daily. This ensures the proper alignment of the isocenter system in relation to the linear accelerator isocenter. In this case, a special phantom (ET isocenter phantom) is used, measuring 10 × 10 cm^2^, with five reflective marker spheres attached to its upper surface^(6)^. To calibrate the X-rays, another special phantom must be used. That one, in turn, ensures precision when correcting and checking positioning during treatment.

Both tests were carried out in order to work with residual errors with an absolute value of ≤ 1 mm, according to the manufacturer's recommendations.

The positioning of the X-ray calibration isocenter in relation to the mechanical isocenter of the linear particle accelerator is checked using a Winston-Lutz phantom, which comprises a small metallic sphere composed of steel, titanium or tungsten, inserted onto the end of a staff made of the same material, representing the treatment target. The difference between the center of the sphere, its projection, and the center of the treatment field reveals the isocenter movement, which should be ≤ 0.7 mm for stereotactic treatments.^(6)^

### The ART-210 and the hidden target test

The hidden target test is used in order to evaluate the system accuracy for radiosurgery, whether frame-based or frameless. This test requires a head phantom^(6)^. Metallic marker spheres are placed inside the phantom to simulate possible lesions. It is thus possible to analyze the precision with which the spheres are detected. As a result, setup and residual errors (after due corrections) are obtained for the system in question.

For this study, an ART-210 anthropomorphic head phantom was used. This phantom is composed of urethane with effective atomic number and mass density similar to those of the muscle, adipose tissue and bone typical of each region, with 2.5 cm-thick cross sections.

**Figure 1:**
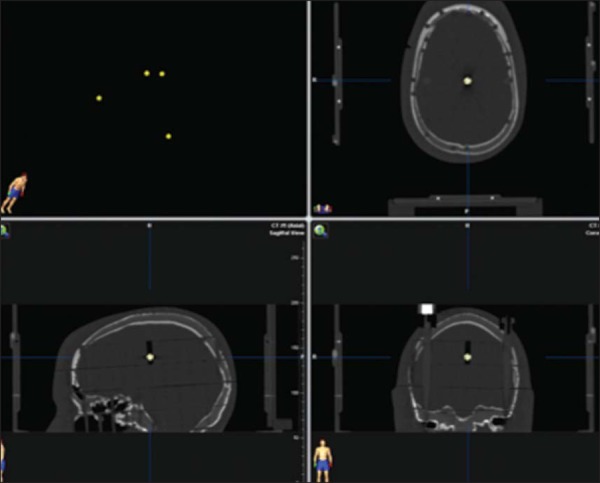
Tomography images of the ART-210 anthropomorphic head phantom with the four metallic marker spheres highlighted.

Four 5-mm diameter metallic spheres were placed inside four different cross sections of the head phantom, in order to simulate brain lesions. The four spheres were placed inside the frontoparietal lobe, the frontal lobe, the temporo-occipital lobe, and the base of the skull, respectively (Figure 1).

In both methods, the same tools used in a real procedure were employed. Adhesive strips were used in order to avoid movement between sections of the head phantom.

CT slices (0.625 mm-thick) were obtained with the head phantom properly positioned for the frameless radiosurgery and, subsequently, for the frame-based radiosurgery, on a flat table and with the appropriate laser system.

### Frameless radiosurgery

**Figure 2:**
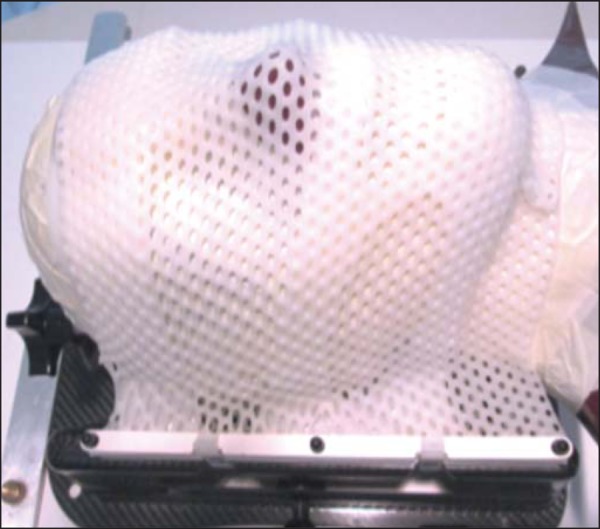
ART-210 head phantom properly positioned on the support base, with the set of frameless masks adequately molded to its cranial surface.

For the frameless radiosurgery, a system of thermoplastic masks (iPlan RT; Brainlab AG) molded the cranial surface of the phantom. All parts of the system were previously bathed in water at 70ºC for approximately 5 minutes, according to the manufacturer's recommendations. To make the mold, the anthropomorphic head phantom was properly positioned upon a special nonrotating support base. Each mold was placed individually and maintained over the skull for approximately 30 minutes, until the transparent plastic surface became opaque and totally dry (Figure 2). During the acquisition of the planning images, an acrylic locating box was linked to the frameless system support base. Fiducial markers placed in the box allowed the phantom cranial structures to be correlated with the data coordinates imported into the iPlan RT system^(6)^.

### Frame-based radiosurgery

**Figure 3:**
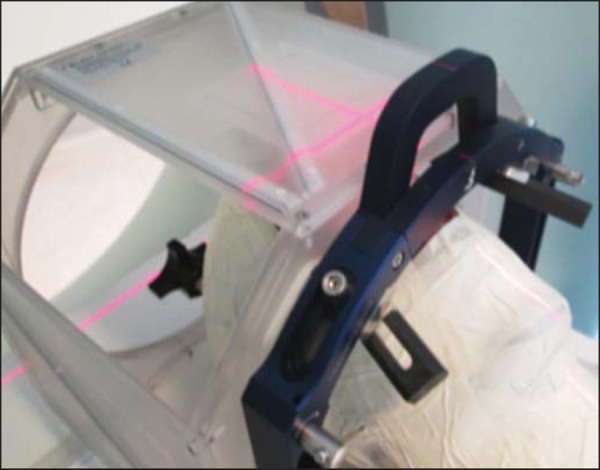
CT images of the ART-210 head phantom properly positioned on the support base with the frame-based radiosurgery apparatus.

For the frame-based radiosurgery, the cranial halo (Brainlab AG), duly fixed in the supine position, without cranial rotation, was used. The halo angle in relation to the interorbital line was 0º to 10º. A 210 mm long locating box was attached to the cranial halo during the imaging process (Figure 3). This tool sets the cranial volume with stereotactic coordinates 3D precision and determines the exact position of the geometric matrix of the structures in question^(6)^.

### ExacTrac verification

After the aforementioned quality control tests, the CT images for frame-based radiosurgery and frameless radiosurgery were registered in the treatment program.

An infrared reader identifies the reflective marker spheres attached to an apparatus fixed on the treatment table. The location of the spheres is geometrically correlated to the isocenter indicated by the planning CT.

Usually, the ExacTrac system is not used in frame-based radiosurgery. To analyze the setup errors identified by this system, it was necessary to use an alternative technique for positioning the apparatus with the reflective spheres, in order to attach them to the stereotactic halo, thus allowing corrections and verifications.

With the ExacTrac system, it is possible to establish a maximum tolerance in relation to the residual error resulting from every table movement or change in the treatment isocenter^(6)^. The absolute (in module) tolerance in this study was 1 mm.

Error analyses were always made with table and gantry at 0º, for frame-based and frameless techniques.

## RESULTS

Information related to setup errors and residual errors in each isocenter was collected for all three translational directions: lateral, longitudinal, and vertical.

**Figure 4:**
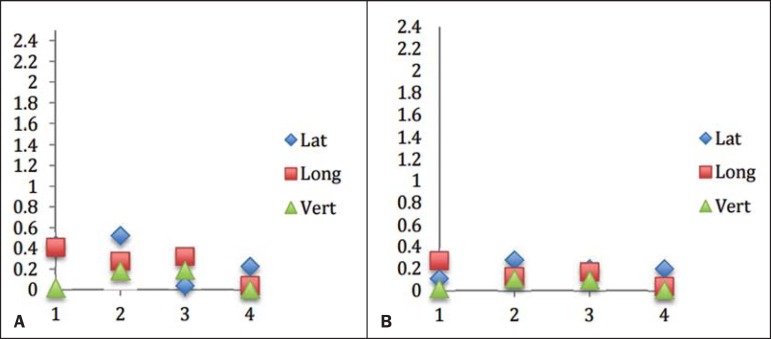
Frameless radiosurgery errors for the four previously created isocenters, in the three translational directions. Coordinate x refers to the four isocenters, and coordinate y refers to the resulting error values, in millimeters. A: Setup errors. B: Residual errors.

**Figure 5:**
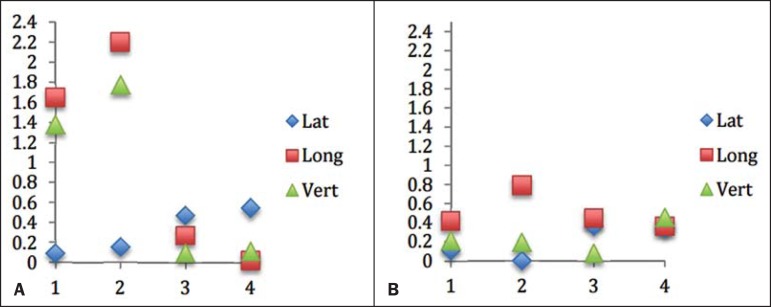
Frame-based radiosurgery errors for the four previously created isocenters, in the three translational directions. Coordinate x refers to the four isocenters, and coordinate y refers to the resulting error values, in millimeters. A: Setup errors. B: Residual errors.

Figures 4 and 5 show the results obtained for each isocenter in each translational direction, in absolute values.

**Figure 6:**
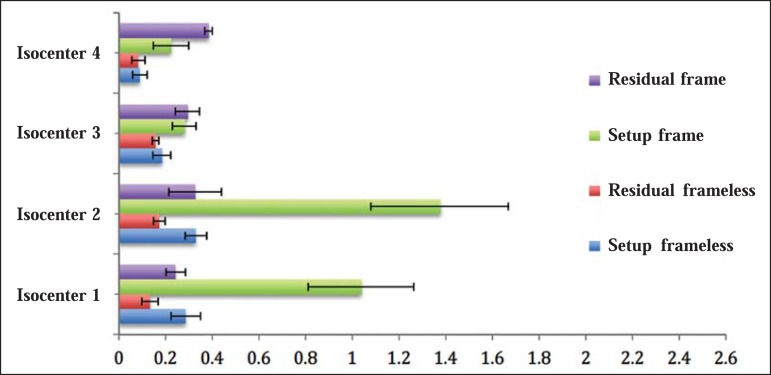
3D translational setup errors in frame-based radiosurgery and frameless radiosurgery for the four isocenters separately. Coordinate x refers to the resulting values and coordinate y refers to the isocenters in question. The error bar shows the average standard deviation for each set of results.

Figure 6 shows the absolute average values, relative to the setup and translational residual errors considering the three directions, for each radiosurgery method, per treatment isocenter.

For the frameless technique, the average magnitude (considering the four isocenters) of translational setup errors was 0.22 ± 0.04 mm, with a combined uncertainty of 0.28 mm, calculated according to the propagation of uncertainty theory. The translational residual errors average value was of 0.14 ± 0.02 mm, with a combined uncertainty 0.16 mm.

For the frame-based technique, the average translational setup error magnitude was 0.73 ± 0.14 mm, with a combined uncertainty of 1.15 mm. The average magnitude of the translational residual errors was 0.31 ± 0.04 mm, with a combined uncertainty of 0.63 mm.

## DISCUSSION

Radiosurgery plays an important role in the treatment of intracranial lesions. Precise techniques for locating the target volumes that need treatment, as well as adequate patient immobilization, require multiple tests of the methods employed. Rigorous, daily checks of the treatment systems and the correct use of the apparatus used in these procedures contribute to a more effective result^(5,6)^.

The current literature shows that there have been great medical advances in the use of IGRT systems. In relation to radiosurgery, the frameless method avoids the discomfort issues encountered with the frame-based method and allows the treatment to be fractionated^(3,5,7-14)^.

Our results reveal significant advantages in checking the target position, down to the submillimeter level, during treatment by applying X-ray images. The possibility of realigning the patient during the phase that precedes each irradiation increases the safety of high-dose treatments, such as radiosurgery.

According to Lamba et al.^(3)^, possible error sources during a frame-based radiosurgery include inadequate movement of the stereotactic arc, misalignment of the lasers that compose the system, incoherencies in the collimators and isocenters, whether in the equipment itself or the target volume, and variations in the treatment table. These components may allow subtle movement in the assembly system and, consequently, small final translational deviations. Even so, the frame-based method is considered by many the gold standard in radiosurgery, due to the stability acquired with bone fixation. As a comparative measure, there have been many studies on the precision of these two methods^(3-5,10,11)^.

This study was developed with the purpose of providing more data on the setup errors and residual errors of frame-based and frameless radiosurgery. Therefore, an end-to-end test was carried out using the ART-210 anthropomorphic head phantom. By evaluating the average errors per isocenter, standard deviation of the samples and averages, as well as the combined uncertainties, it was possible to verify that the two methods showed equivalent precision, without evidence of statistically significant differences.

With this study, the efficiency of the ExacTrac system was analyzed regarding the significant setup error reduction after the proper corrections suggested by the system, resulting in the residual errors. For frame-based radiosurgery, those corrections were more noticeable, with differences of up to 1 mm between the setup error and final residual error.

Considering the magnitude of the errors in frame-based radiosurgery and frameless radiosurgery, the greater differences observed in frame-based radiosurgery may have occurred due to small movements of the stereotactic arc after it had been fixed to the anthropomorphic head phantom. This phantom does not have skin tissue, representing a difference in comparison to a real patient.

Although the end-to-end test with anthropomorphic phantoms is widely used in order to determine the precision and technical capacity of these systems, this model is based on ideal conditions. Clinical application represents a more complex challenge for these systems, possibly presenting more reliable results.

Ramakrishna et al.^(5)^ obtained results with a phantom similar to those obtained with real patients, showing that the general precision of the IGRT system is similar to that of frame-based radiosurgery. The intrafraction motion was greater in frameless radiosurgery, although the values remained within an adequate range for the stereotactic treatment. Reflecting on these results, the authors stated that, even though it may be argued that image-guided positioning can be more precise than or equally as precise as frame-based radiosurgery, the greater intrafraction motion should be considered. Said study showed an intrafraction dislocation of up to 2 mm in approximately 22% of patients analyzed during frameless radiosurgery. As a preventive measure, the authors suggested using frames for targets smaller than 5 mm.

In the present study, frameless radiosurgery showed more homogeneous results than did frame-based radiosurgery. However, when we analyzed the general average values shown in Figure 6, isocenter 4 showed smaller values and isocenter 2 had higher values. As described in the section **ART-210 and hidden target test**, isocenter 4 is found at the base of the skull of the phantom, whereas isocenter 2 is in the frontal lobe. Although there is no consensus on the possibility of greater or smaller movement of the target depending on the region in which it is found, hypothetically, it may inferred that lesions that are more frontal deserve greater care because they are subject to greater cranial movement. Some authors have stated that, during approximately two thirds of the treatment time, movement is more frequently registered in the longitudinal or craniocaudal direction^(3)^, exactly the movement which resulted in a higher value for frame-based radiosurgery (see Figure 5). It is possible that the results for frameless radiosurgery were of lesser magnitude in the longitudinal direction because the basal mold was carefully positioned on the cranial support and stretched to the top of the head phantom, thereby increasing stability in that direction.

## CONCLUSION

By studying the average values, standard deviations and combined uncertainties, it was possible to evaluate and compare setup errors and 3D translational residual errors in frame-based and frameless image-guided radiosurgery, using an ART-210 anthropomorphic head phantom. The results showed no evidence of significant differences between the two immobilization methods, suggesting equivalent precision. In addition, the image system employed had very good setup error correction in this test, particularly in frameless radiosurgery, resulting in residual errors close to zero.
